# Cytoplasmic Transport Machinery of the SPF27 Homologue Num1 in *Ustilago maydis*

**DOI:** 10.1038/s41598-018-21628-y

**Published:** 2018-02-26

**Authors:** Lu Zhou, Theresa Obhof, Karina Schneider, Michael Feldbrügge, G. Ulrich Nienhaus, Jörg Kämper

**Affiliations:** 1 Institute of Applied Physics, Karlsruhe Institute of Technology (KIT), Karlsruhe, Germany; 2 Institute of Nanotechnology, Karlsruhe Institute of Technology (KIT), Karlsruhe, Germany; 3 Department of Genetics, Institute of Applied Biosciences, Karlsruhe Institute of Technology (KIT), Karlsruhe, Germany; 4 Institute of Microbiology, Cluster of Excellence on Plant Sciences, Heinrich-Heine-University, Düsseldorf, Germany; 5 Department of Physics, University of Illinois at Urbana-Champaign, Urbana, IL USA; 6 Institute of Toxicology and Genetics, Karlsruhe Institute of Technology (KIT), Eggenstein-Leopoldshafen, Germany

## Abstract

In the phytopathogenic basidiomycete *Ustilago maydis*, the Num1 protein has a pivotal function in hyphal morphogenesis. Num1 functions as a core component of the spliceosome-associated Prp19/CDC5 complex (NTC). The interaction of Num1 with the kinesin motor Kin1 suggests a connection between a component of the splicing machinery and cytoplasmic trafficking processes. Previously it was shown that Num1 localizes predominantly in the nucleus; however, due to the diffraction-limited spatial resolution of conventional optical microscopy, it was not possible to attribute the localization to specific structures within the cytoplasm. We have now employed super-resolution localization microscopy to visualize Num1 in the cytoplasm by fusing it to a tandem dimeric Eos fluorescent protein (tdEosFP). The Num1 protein is localized within the cytoplasm with an enhanced density in the vicinity of microtubules. Num1 movement is found predominantly close to the nucleus. Movement is dependent on its interaction partner Kin1, but independent of Kin3. Our results provide strong evidence that, in addition to its involvement in splicing in the nucleus, Num1 has an additional functional role in the cytosol connected to the Kin1 motor protein.

## Introduction

The phytopathogenic basidiomycete *Ustilago maydis* causes smut disease on corn (*Zea mays*). *U*. *maydis* displays a dimorphic life cycle, with yeast-like, haploid sporidia that divide by budding, and a dikaryotic stage in which the fungus grows as filament. The latter stage is initiated by fusion of two sporidia and initiates the biotrophic phase, in which *U*. *maydis* depends on its host plant for further propagation^1^.

The major control instance for the dimorphic switch consists of a heterodimeric complex of two homeodomain transcription factors, bE and bW, both encoded by the *b*-mating type locus. The heterodimer is formed only if the two proteins originate from two sporidia with different *b*-alleles (e.g. bE1 and bW2). Cells with an activated *b*-pathway grow as filaments, but only the tip compartment that contains the genetically distinct nuclei is filled with cytoplasm, whereas the distal parts of the hyphae are composed of empty segments separated by septa. Only after penetration of the plant surface, “true” filaments with multiple septated compartments are developed reviewed in^1^.

Prerequisite for filamentous growth of the hyphae is the establishment and maintenance of a defined axis of polarity. Polar tip growth is dependent on long-distance transport towards the growth cones at the cell apices, which is facilitated by arrays of microtubules and microtubule-dependent proteins^2^. The cargo transported includes endosomes, peroxisomes, nuclei, but also mRNA, which passively travels on endosomes^3^. The motors required for transport in *U*. *maydis* include the plus-end directed UNC104-like kinesin-3 motor protein Kin3, which moves endosomes in both directions within the cell along an array of antiparallel microtubules^4,5^. The minus-end directed dynein motor protein Dyn1/2^4–7^ is particularly important at the poles of filaments, where unipolar microtubules with their plus ends extend from the antiparallel array^6,8^. The kinesin motor protein Kin1 contributes to hyphal morphology by transporting dynein in the direction of the microtubule plus ends within the hyphal apex, where a loading zone for retrograde transport processes is established^6,8^. Kin1 is also involved in organelle transport^9^, to foster transport of secretory vesicles into the growing tip^10^, or to deliver specific cargo proteins such as the fungal-specific class-17 myosin Mcs1^11–13^.

We have recently identified the Num1 protein, which has a pivotal function in hyphal morphogenesis^14^. Num1 is homologous to SPF27, a core component of the evolutionarily conserved Prp19/CDC5 complex (NTC), which is an integral component of active spliceosomes and required for intron removal during pre-mRNA splicing^15^. In addition to regulating spliceosome formation and splicing fidelity, the complex has a conserved function in cellular response to DNA damage and cell cycle checkpoint control^16–24^.

Hyphae of *num1* deletion strains exhibit pleiotropic polarity defects and, in line with the described NTC functions, the *num1* mutation affects cell cycle regulation and survival after UV irradiation. In addition, the *num1* deletion leads to reduced splicing efficiencies on a global scale. Num1 was shown to interact with two conserved core components of the NTC complex, Cdc5 and Prp19^14^. However, also several proteins with putative functions during vesicle-mediated transport processes were identified as potential Num1 interactors in a yeast two-hybrid screen; in particular, the kinesin 1 motor protein Kin1 was shown to physically interact with Num1^14^. Overlapping phenotypes with respect to altered polar growth, altered vacuolar morphology, dynein localization as well as loss of motility of early endosomes (EEs) further corroborate the interaction of Num1 and Kin1^14^. Taken together, these data implicate a connection between a component of the splicing machinery and cytoplasmic trafficking processes. As the *num1*-mutation also affects cytoplasmic mRNA transport, the protein may constitute a novel functional interconnection between the two disparate mechanisms of splicing and trafficking. According to our model, the Num1 protein functions in the coordination of pre-mRNA splicing with nuclear pore complex-dependent export of mRNP particles and microtubule-based mRNA transport.

Our previous studies have shown that the Num1 protein localizes predominantly in the nucleus^14^. In accordance with its cytoplasmic function, we have also provided evidence of cytoplasmic localization of Num1:GFP fusion proteins. However, due to weak signals and the diffraction-limited spatial resolution of conventional optical microscopy, it was not possible to attribute the localization of Num1:GFP fusion proteins to specific cytoplasmic structures or organelles.

In this work, we have employed super-resolution localization microscopy (photo-activation localization microscopy, PALM/stochastic optical reconstruction microscopy, STORM^25–27^) to clearly visualize Num1 localization in the cytoplasm. PALM is a widefield imaging technique that is based on the detection of individual, photoactivable fluorescent proteins^28^. Upon light absorption, these proteins, can either switch from a dark to a fluorescence emitting state or change their absorption and emission color^29^. Within each camera frame, only a small number of individual fluorophores are stochastically photoactivated and precisely localized by sophisticated algorithms such as a-livePALM^30^. Subsequently, super-resolved images are reconstructed from a large stack of sequential camera images. Here, we have adapted a tandem dimeric version (tdEosFP^31^) of EosFP, a tetrameric fluorescent protein isolated from the coral *Lobophyllia hemprichii*^32,33^, as a photoactivatable probe for PALM in *U*. *maydis*. EosFP changes its emission color irreversibly from green to red upon irradiation with violet light. Our new data employing Num1:tdEosFP fusion proteins reveal that Num1 is localized within the cytoplasm and enhanced in the vicinity of microtubules. It does, however, not colocalize with EEs. Num1 motility is Kin1 dependent, but independent of Kin3. These results provide strong evidence that, in addition to its involvement in splicing in the nucleus, Num1 has an additional functional role in the cytosol connected to the Kin1 motor protein.

## Results

### Establishment of a strongly fluorescent EosFP for super-resolution microscopy in *U. maydis*

The interaction with the motor protein Kin1 observed in the yeast two-hybrid screen implicates a cytoplasmic localization of Num1. Previously, we detected cytoplasmic signals from a Num1:3GFP fusion protein with conventional fluorescence microscopy, however, fluorescent signals were too weak to assign them to specific cellular structures. In this study, we aimed at using PALM to benefit from its single-molecule sensitivity and capability of localizing emitters with nanoscale resolution. To this end, we constructed fusion proteins of Num1 with EosFP, which is a tetrameric protein in its natural form. Because oligomerization is often detrimental in imaging applications requiring functional fusion constructs, dimeric and monomeric EosFP variants have been generated by protein engineering^34^. For example, the change of the amino acid 158 from threonine to arginine (T158R) causes a splitting of the EosFP tetramer into dimers^31^. Other amino acids have been replaced to enhance stability or reduce the oligomerization tendency^31,35^. We have started our efforts with the frequently used photo-convertible fluorescent protein mEos2, a monomeric variant of EosFP that expresses well at 37 °C^36^. The mEos2 open reading frame was dicodon optimized for expression in *U*. *maydis*^37^ and fused to the 3′ end of the *num1* gene. The construct was integrated into the *num1* locus of strain AB31 by homologous recombination to express the *num1:mEos2* fusion gene under the native promoter of *num1* in its natural context. The *U*. *maydis* strain AB31 (*a1 P*_*crg1*_*:bE1/P*_*crg1*_*:bW2*) harbors a set of compatible *bE1* and *bW2* genes under control of the arabinose-responsive *P*_*crg1*_ promoter^38^. In glucose-containing media, AB31 grows yeast-like, but upon arabinose-induced expression of *bE1/bW2*, the strain switches to polarized growth and forms long filaments reminiscent to those formed after fusion of compatible sporidia^38,39^.

**Figure 1:**
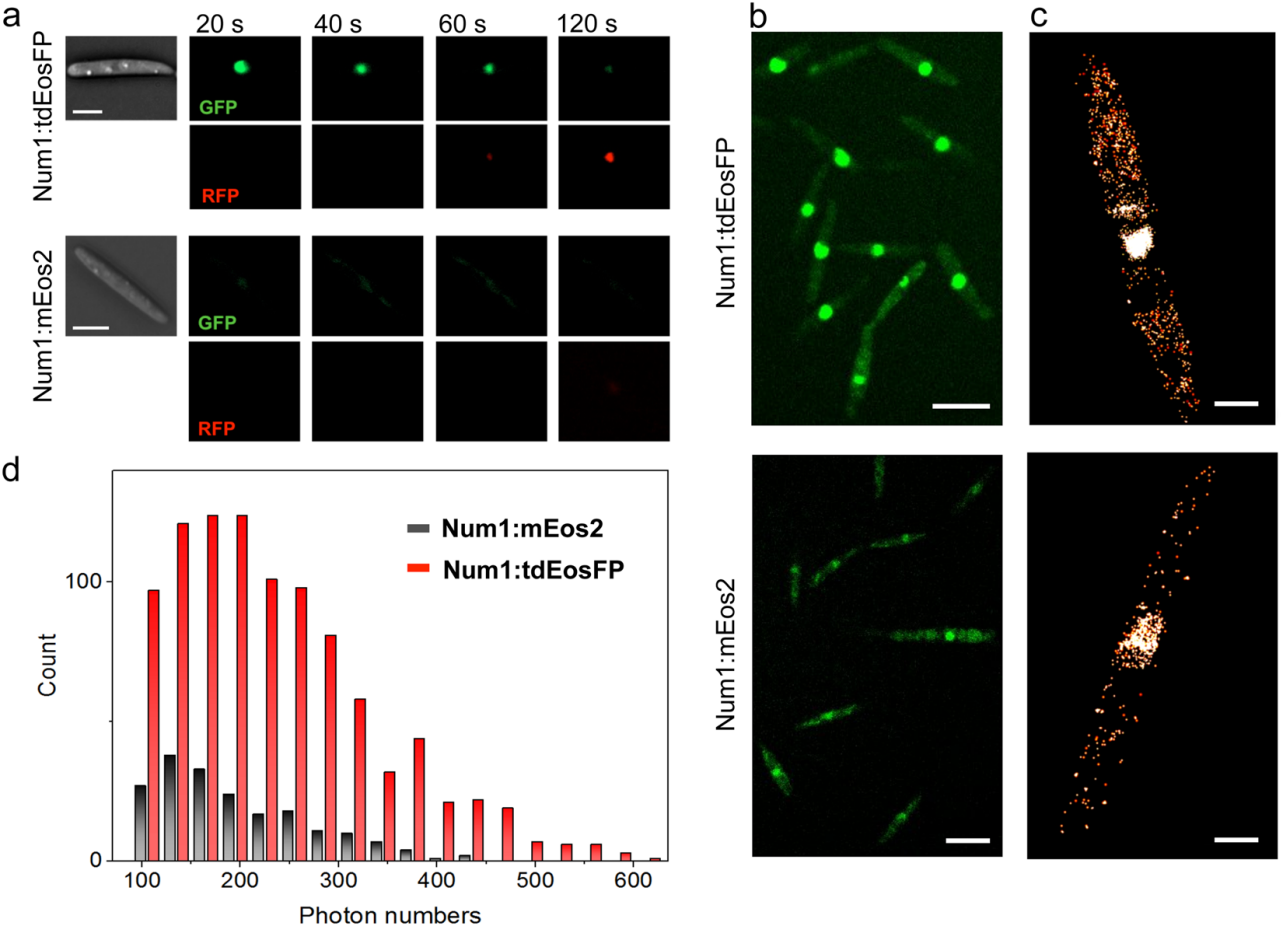
Comparison between Num1:tdEosFP and Num1:mEos2. *U*. *maydis* AB31 sporidia expressing Num1:tdEosFP (upper panels) and Num1:mEos2 (lower panels), both under control of the endogenous *num1* promoter, were imaged with different microscopy techniques to visualize Num1 fusion constructs with different fluorescent proteins. (**a**) Widefield fluorescence microscopy. EosFP was photoconverted with light of ~365 nm wavelength in 20 s intervals; after each interval, red and green emitting species were imaged. Scale bars: 5 µm. (**b**) Confocal microscopy of the green EosFP species of the EosFP variants, with both images adjusted to the same intensity contrast to allow a direct comparison of the different EosFP variants. Scale bars: 10 µm. (**c**) Localization microscopy (PALM). Images were reconstructed from 1000 camera frames, using a photon number threshold of 100, scale bars: 2 µm. (**d**) Photon number distributions of fluorescence events collected from the cytoplasmic regions of the two cells shown in C, showing the number of localization events as a function of the registered photons per frame for each event.

The fluorescence signal of AB31 cells expressing Num1:mEos2 was monitored by conventional widefield fluorescence microscopy. EosFPs were photoconverted using a filter set with maximum transmission at 365 nm; fluorescence of the red and green species was measured in 20 s intervals. Only weak fluorescence in the green channel was detected from the nucleus; in contrast to our data obtained with a Num1:3GFP fusion protein^14^, cytoplasmic signals were not visible (Fig. 1a, upper panel). Conversion to the red EosFP species was not detectable within a time frame of 120 s. Thus, mEos2 does not appear to be feasible for PALM microscopy in *U*. *maydis*.

One option to increase the signal is to express the fusion protein by means of a strong promoter. However, as overexpression might lead to artificial localization, we aimed to increase the sensitivity of the EosFP probe. To this end, we constructed a tandem dimeric EosFP fusion protein optimized for use in *U*. *maydis*. We introduced the T158R mutation that splits the tetramer into dimers into a dicodon optimized EosFP open reading frame, and tethered two copies of the gene via a linker sequence encoding 12 amino acids^31^. We did not introduce a mutation to increase the thermostability because the dimeric as well as the monomeric EosFP proteins both express well at the optimal growth temperature of *U*. *maydis* (28 °C)^34^. In analogy to mEos2, the tandem dimeric EosFP (tdEosFP) open reading frame was fused in frame to the 3′ end of the *num1* gene and introduced into the *num1* locus of AB31.

Conventional fluorescence microscopy revealed brighter signals for Num1:tdEosFP than for Num1:mEos2. The emission in the green channel (prior to photo-conversion) was much brighter in sporidia of AB31 *num1:tdEosFP* than in AB31 *num1:mEos2*; Num1:tdEosFP was converted within 120 s to the red species (Fig. 1a). Likewise, in images acquired using confocal microscopy to visualize the green EosFP species, Num1:tdEosFP appeared significantly brighter than Num1:mEos2 (Fig. 1b).

### PALM microscopy reveals cytoplasmic localization of Num1

As the novel tdEosFP performed well with respect to both fluorescent signal intensity as well as photoconversion, we next used PALM microscopy to localize Num1 EosFP fusion proteins in AB31 sporidia expressing Num1:tdEosFP; for comparison, we also studied AB31 expressing Num1:mEos2. PALM images, rendered from 1000 successive camera frames each, were acquired with 561 nm laser excitation and an additional weak 405 nm laser irradiation for continuous green-to-red photoconversion of the fluorescent proteins; the emission was filtered with a 607/70 band-pass filter. A photon number threshold of 100 was set for collecting fluorescent events in the red channel. As expected, both strains showed maximum fluorescence from the nucleus. However, fluorescence signals were also detectable within the cytoplasm (Fig. 1c), indicating a dispersed cytoplasmic localization of Num1. Both the number of detected fluorescent proteins as well as their intensities were much greater in AB31 sporidia expressing Num1:tdEosFP than in those expressing Num1:mEos2. The histograms given in Fig. 1d display the number of localization events as a function of the registered photons per frame for each event. For Num1:tdEosFP, the average number of photons from 965 events was 236; for Num1:mEos2, it was only 198 from 192 events. These data underscore that the tdEosFP fusion construct is significantly superior to the one with mEos2 for super-resolution imaging of proteins.

**Figure 2:**
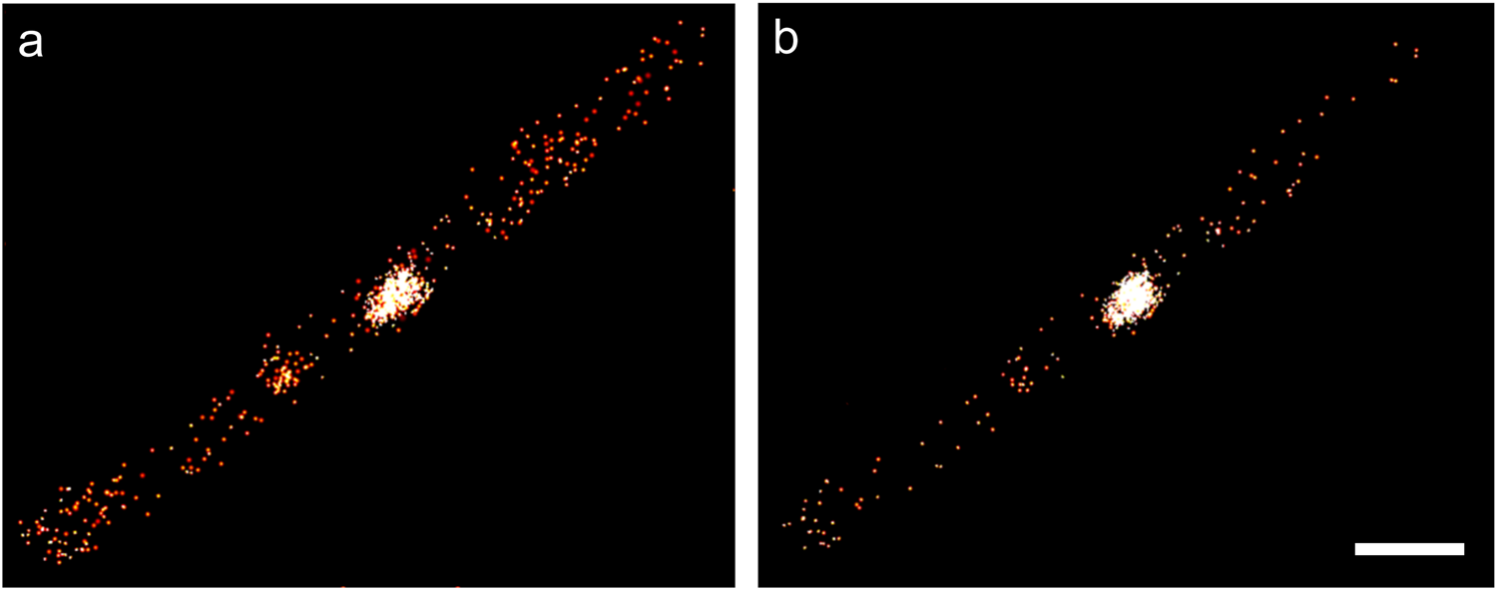
PALM Super-resolution imaging of an AB31 sporidium expressing Num1:tdEosFP. The images were rendered from the same raw data, with photon number threshold set to (**a**) 100 and (**b**) 300. Scale bar, 2 µm.

The localization precision of a fluorophore scales approximately with the square root of the number of photons collected during exposure. Effects such as fluorophore movement or a slight defocus may lead to lower photon numbers collected from an individual molecule and, therefore, less accurate localization. Such events can be excluded by setting a proper photon threshold; events below this threshold will simply be discarded. Taking a high photon number threshold in image reconstruction leads to a smaller number of events, but selective inclusion of very precisely localized events. Consequently, the image quality improves as long as the number of localization events is still sufficiently high. In Fig. 2, we compare a localization microscopy data set rendered with photon number thresholds of 100 (Fig. 2a) to 300 (Fig. 2b). Under these more stringent imaging conditions, the Num1:tdEosFP protein is still clearly dispersed throughout the entire cytoplasm.

Both the interaction with the microtubule-associated motor protein Kin1 as well as the finding that deletion of *num1* affects the movement of EEs, which are transported via a microtubule-dependent machinery^14^, suggest that Num1 might be associated with either microtubules and/or EEs. To address a possible connection between Num1 and the microtubule cytoskeleton, we expressed a GFP:Tub1 fusion protein^40^ ectopically in AB31 *num1:tdEosFP* to visualize microtubules. Cells were sequentially imaged in green (*GFP:tub1*) and red (*num1:tdEosFP*) channels, and images were subsequently merged (see Materials and Methods).

**Figure 3:**
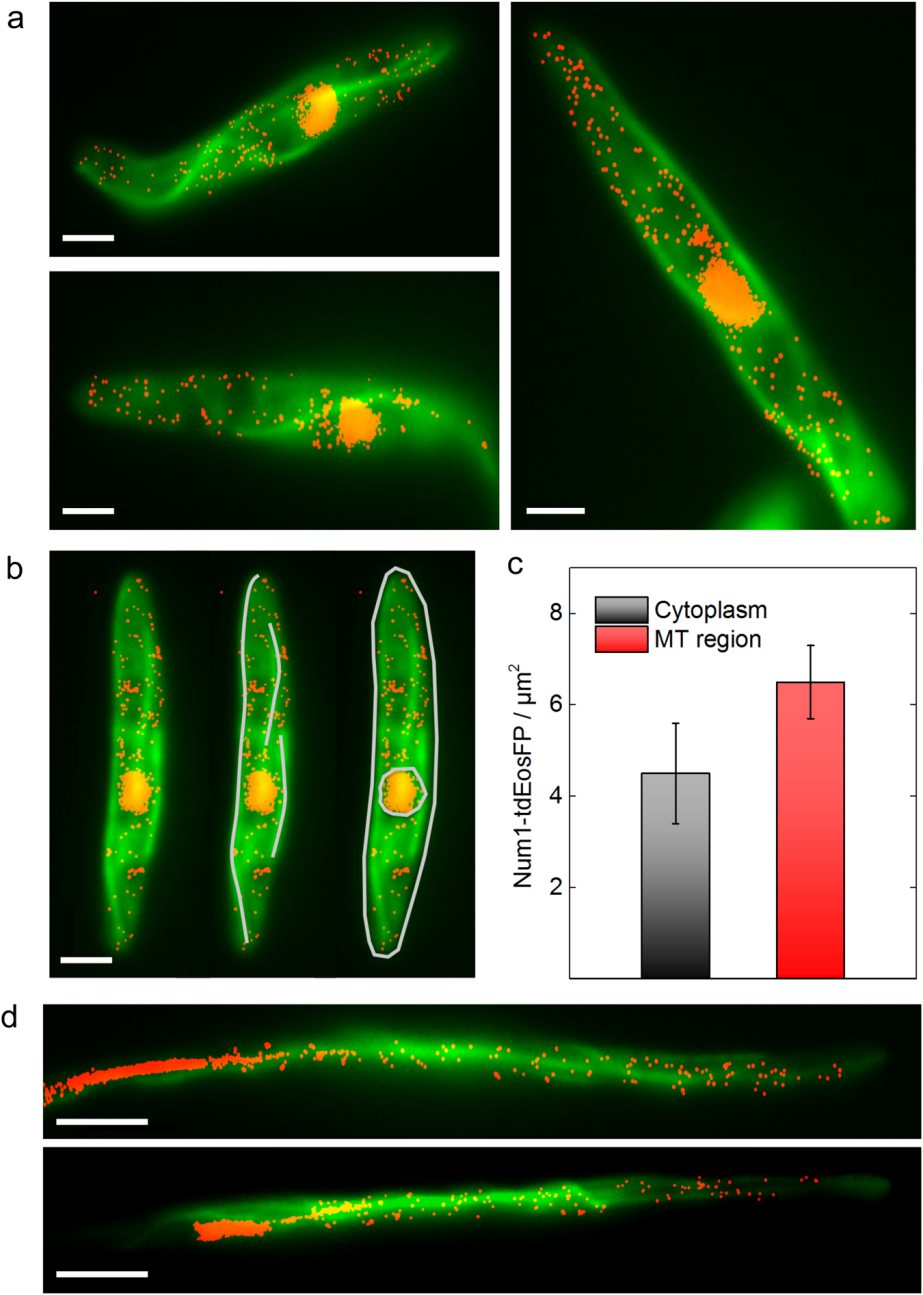
Elevated Num1 localization in the vicinity of microtubules. Num1:tdEosFP (red) was co-expressed with the tubulin marker GFP:Tub1 (green) in strain AB31 and analyzed in sporidia; Num1:tdEosFP was expressed under its natural promoter, GFP:Tub1 under the strong P_*otef*_ promoter ectopically integrated in the *ip*-locus. (**a**) Two-color image displaying the fluorescence from the microtubule structure (GFP:Tub1) and individual Num1:tdEosFP proteins. Molecule positions of Num1:tdEosFP were analyzed and rendered to yield the super-resolved localization image overlaid with the regular image from the green channel. Scale bar: 2 µm. (**b**) Illustration showing how images (left) were processed to define microtubule (middle) and cytoplasmic regions (right) for Num1:tdEosFP density calculations given in C; signals from the nuclear region were excluded in these calculations. Scale bar: 2 µm. (**c**) Density of Num1:tdEosFP molecules in the cytoplasm and microtubule regions (mean ± SD, n = 9). (**d**) Two-channel images of AB31 cells expressing GFP:Tub1 and Num1:tdEosFP. Cells were grown in arabinose containing medium for 6 h to induce filament formation. Emission from Num1:tdEosFP (red) is seen within the entire cytoplasm and partially associated with microtubules. Scale bars: 5 µm.

In AB31 sporidia, Num1:tdEosFP shows partial colocalization with GFP-labeled microtubules; however, tdEosFP signals are also visible in regions distinct from microtubules (Fig. 3a). We next questioned whether Num1 might localize predominantly in the vicinity of microtubules, and calculated the density of Num1:tdEosFP (1) in the microtubule region and (2) the cytoplasmic region of the entire cell. Based on microtubule images in the green channel, lines were drawn by hand to trace the microtubules, and the microtubule region was defined as those pixels that were closer than 300 nm (12 pixels with 25 nm pixel size) to the center of the lines (Fig. 3b, middle panel). The ±300 nm tolerance was chosen to account for possible movements of microtubules during imaging in the red channel (1000 frames, 50 s). As a control, the density of Num1:tdEosFP localization events was also calculated for the whole cytoplasmic region, i.e., the entire cell without the nuclear region (Fig. 3b, right panel). Averaged over nine cells, we obtained 6.5 ± 0.8 µm^−2^ for the microtubule region, which is significantly larger than the value of 4.5 ± 1.1 µm^2^ for the cytoplasmic region (Student’s t-test, p < 0.0005) (Fig. 3c). This quantitative analysis supports the notion that Num1 is partially co-localized with microtubules.

To address the localization of Num1 in filamentous cells, we induced the expression of *bE1* and *bW2* in AB31 for 6 h in arabinose-containing medium. The resulting hyphae do not show any obvious phenotype with respect to morphology, septum formation or nuclear distribution, as described previously for *num1* deletion strains^14^, indicating that the Num1:tdEosFP fusion protein is functional. Similar to our observations in sporidia, the main signal of Num1:tdEosFP resides within the nucleus, visible as a stretched, elongated structure in *U*. *maydis* hyphae^14^. Close to the nucleus, Num1:tdEosFP shows linear distributions (Fig. 3d); similar to the observations in sporidia, additional signals are distributed over the entire cytoplasm and are not restricted only to microtubules.

### Localization and movement of Num1 depends on the integrity of the microtubule cytoskeleton

**Figure 4:**
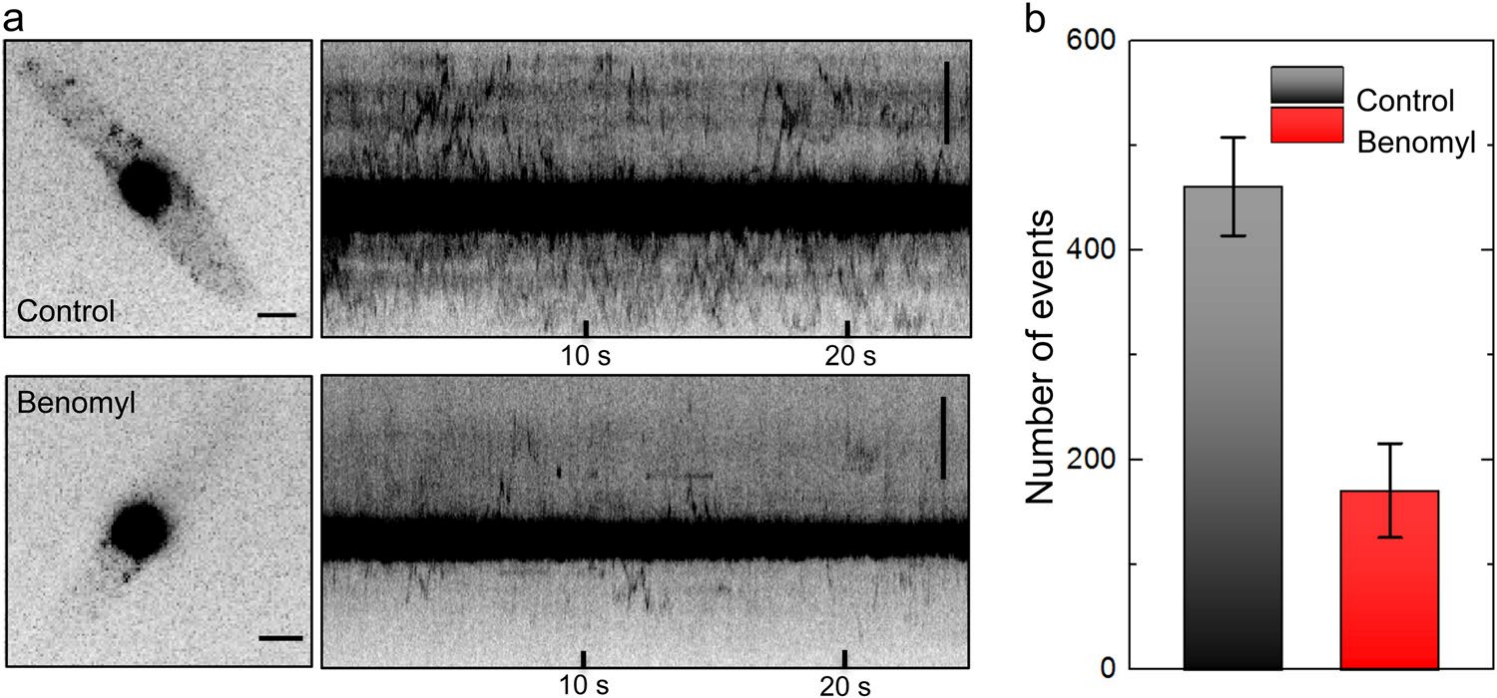
Cytoplasmic movement of Num1:tdEosFP depends on intact microtubules. (**a**) Left: Images of AB31 sporidia expressing Num1:tdEosFP, untreated control (top), and treated with 40 µM benomyl (bottom), a drug disrupting the microtubule cytoskeleton, for 5 min (lower panel); scale bar, 2 µm. Right: Corresponding kymographs, depicting the spatial position of Num1:tdEosFP spots within the cell over time. Kymographs were calculated by integrating over the long axis of the cells, scale bars, 5 µm. The kymograph of wt cells clearly shows cytoplasmic motility of Num1:tdEosFP. Treatment with benomyl leads to a marked reduction of both cytoplasmic localization and motility. (**b**) Number of Num1:tdEosFP molecules in the cytoplasm of control and benomyl treated cells (mean ± SD, n = 8). Data were analyzed from 500 continuous frames with a photon number threshold of 100.

To answer the question whether the Num1 protein moves within the cytoplasm, kymographs were generated along a straight line tracing the long axis of the cells (see Materials and Methods). As shown in Fig. 4a (upper panel), the Num1:tdEosFP protein showed bi-directional movement; proteins travel from the nucleus towards the sporidial tips and back towards the nucleus.

The influence of the microtubule cytoskeleton on the observed Num1 movement in the cytoplasm was analyzed by treatment of cells with the microtubule-destabilizing drug benomyl^41^. Widefield images revealed that the signal of Num1:tdEosFP in the cytoplasm was significantly reduced in the presence of 40 µM benomyl (Fig. 4a, lower panel). For quantification, 500 continuous frames were analyzed for cytoplasmic Num1:tdEosFP signals with the photon number threshold set to 100. 461 ± 47 events (N = 8) were detected for the control cells; for benomyl treated cells, the number of events decreased to 171 ± 45 events (N = 8) (Fig. 4b).

The kymographs of benomyl treated sporidia (Fig. 4a, lower panel) show markedly reduced transport traces of Num1:tdEosFP in the cytoplasm. Some signals in benomyl treated cells show horizontal lines, indicating that Num1 molecules are immobilized as a result of the disintegrated microtubule cytoskeleton. Thus, the integrity of the microtubule cytoskeleton is required for both cytoplasmic localization as well as cytoplasmic movement of Num1.

### Kinesin 1 but not Kinesin 3 is involved in Num1 transport

The requirement for an intact microtubule cytoskeleton suggests that cytoplasmic localization and/or mobility might depend on microtubule-dependent motor proteins, which would be in line with the previously observed interaction of Num1 with Kin1^14^. Accordingly, we assessed the involvement of the two microtubule-associated motor proteins Kin1 and Kin3 on Num1 trafficking. *kin1* and *kin3* were replaced in AB31 *num1:tdEosFP* with a nourseothricin-resistance marker gene by homologous recombination. In the resulting deletion strains AB31 *num1:tdEosFP∆kin1* and AB31 *num1:tdEosFP∆kin3*, movement of Num1:tdEosFP was analyzed in hyphae via kymographs. We focused on hyphae because both the phenotype of *kin1* or *kin3* as well as of *num1* deletions is more prominent in the filament. Deletion of either *kin1* or *kin3* leads to an altered hyphal morphology in AB31; induction of *bE1/bW2* in AB31 wildtype cells results in long filamentous hyphae that originate at one cell pole of the sporidium; in *∆kin1* and *∆kin3* deletion strains, the filament is initiated on both cell poles (bipolar), and the hyphae are much shorter^10^. The deletion also affects shape and localization of the nucleus: in AB31 hyphae, the nucleus migrates into the filament and has a long, stretched appearance, while in *∆kin1* and *∆kin3* strains nuclei remain in the sporidia and are round-shaped.

**Figure 5:**
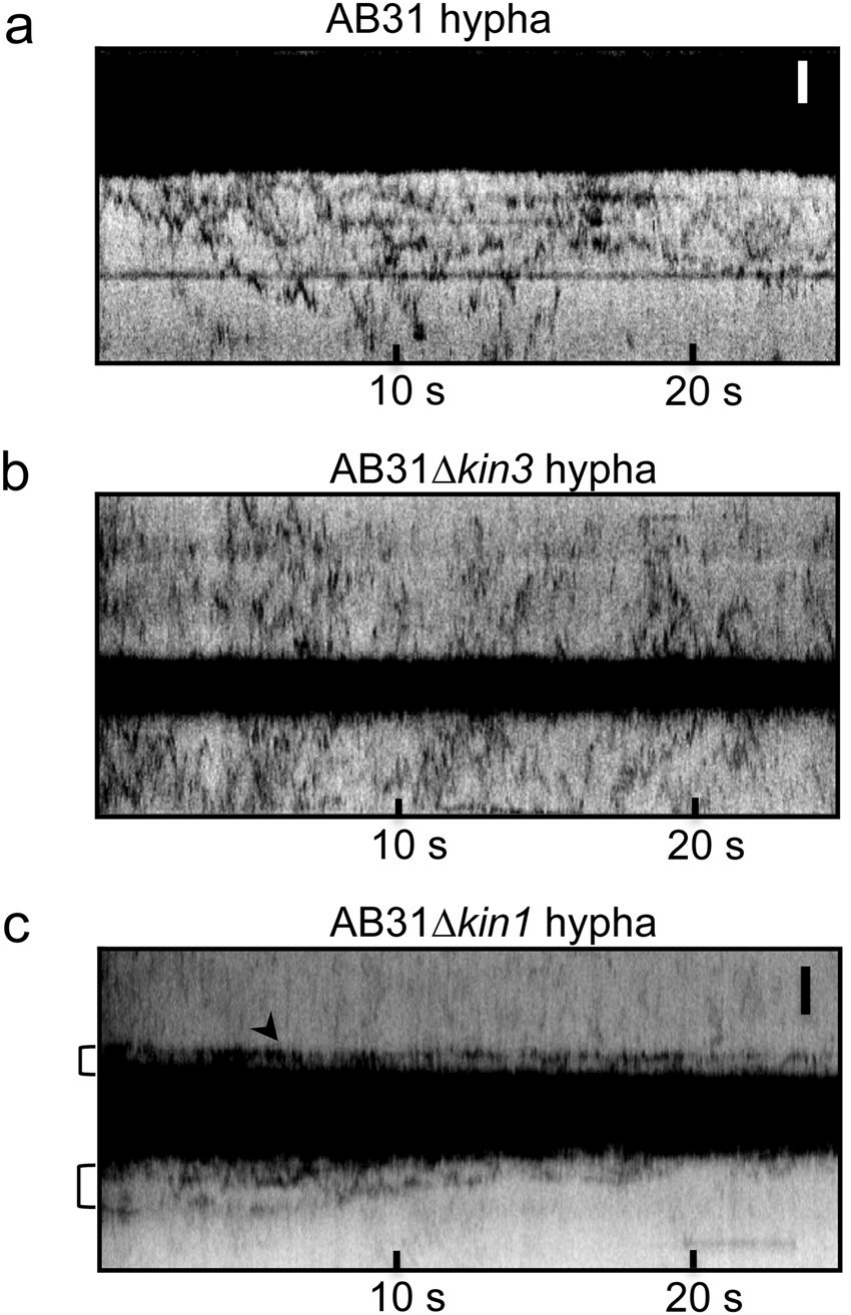
Motility of Num1:tdEosFP in hyphae depends on Kin1 but not on Kin3. Kymographs of AB31 induced hyphae expressing Num1:tdEosFP as well as *kin1* or *kin3* deletion derivatives. All samples were treated identically. Solid black areas represent the nucleus. (**a**) AB31 Num1:tdEosFP. The fusion protein is found to move bidirectionally in the cytoplasm. (**b**) AB31 Num1:tdEosFP ∆*kin3*. The *kin3* deletion has essentially no impact on Num1 motility. (**c**) AB31 Num1:tdEosFP ∆*kin1*. Deletion of *kin1* restricts movement of Num1 to a region close to the nucleus (brackets). Hyphae were induced by arabinose incubation for 6 h. Scale bars: 2 µm.

Although Num1 appears distributed within the entire hypha of AB31 wildtype strains (Figs 3d and 5a), we observe most movement of Num1:tdEosFP in the vicinity (8–10 µm) of the nucleus (Fig. 5a). Deletion of *kin3* does not alter movement of Num1; similar to the situation in AB31 wildtype hyphae, movement is observed around the nucleus (Fig. 5b). In contrast, cytoplasmic movement is nearly completely abolished AB31*∆kin1* filaments: the Num1 molecules are apparently trapped close to the nucleus and cannot move further into the cytoplasm (Fig. 5c).

### Num1 does not co-localize with early endosomes

**Figure 6:**
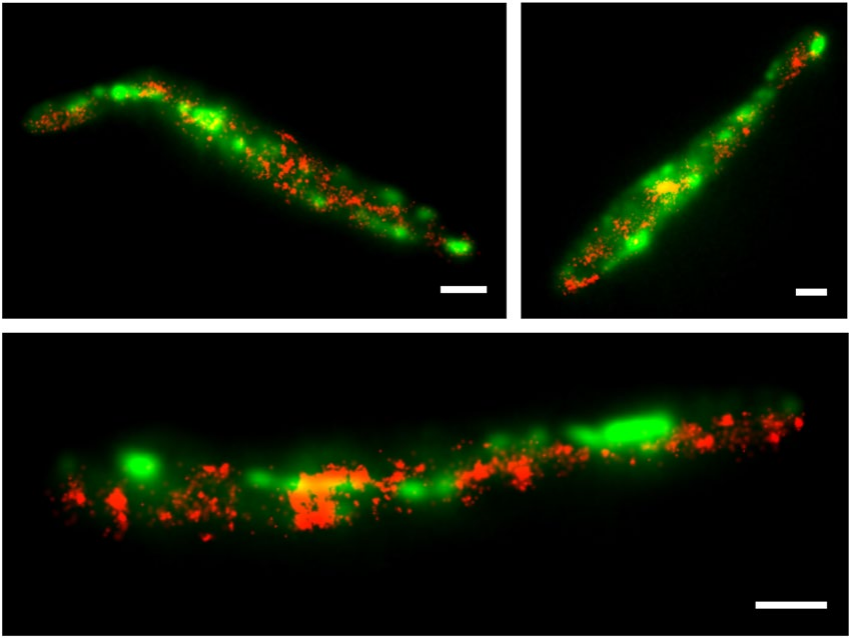
Num1 is not associated with early endosomes. AB31 sporidia expressing Num1:tdEosFP (red) and GFP:Rab5 (green) as a marker for EEs were fixed in 8% formaldehyde and imaged. The widefield image of GFP:Rab5 (green) is overlaid with the localization microscopy image of Num1:tdEosFP (red) in the red channel. There is no co-localization of Num1:tdEosFP with GFP:Rab5 on EEs. Scale bar: 2 µm.

As the deletion of Num1 affects the movement of EEs^14^, we addressed a potential co-localization of Num1 with these vesicles by expressing a GFP:Rab5a fusion protein as a specific marker of EEs^42^ in AB31 *num1:tdEosFP*. Localization microscopy requires a large number of camera frames; thus, the technique is not fast enough to image Rab5a movement in living cells. Therefore, we fixed samples with formaldehyde to study co-localization. GFP:Rab5a labeled EEs are distributed throughout the cytoplasm (Fig. 6); the frequently observed linear array of their localization is in accordance with their microtubule association. However, we did not find co-localization between the GFP:Rab5a and Num1:tdEosFP signals, indicating that Num1 does not localize to EEs.

## Discussion

In our previous study^14^ we failed to localize the Num1 protein to specific structures within the cytoplasm due to its low abundance. We therefore employed PALM to improve both single-molecule sensitivity and resolution of Num1 fusion proteins. Frequently, the monomeric mEos2 protein is used as a fluorescent probe for PALM, in *U*. *maydis*, however, mEos2 showed only a weak fluorescence emission that limited the use for PALM. To improve EosFP as fluorescent probe for *U. maydis*, we constructed a pseudo-monomeric EosFP tandem construct consisting of two protomers connected via a flexible linker engineered so as to favor dimerization. The usefulness of such a tandem dimer construct has been shown previously in HEK293 cells^31,34^. Our data show that tdEosFP is superior to the monomeric mEos2 with respect to sensitivity and brightness: in a direct comparison with a mEos2 fusion protein, about the 5-fold number of molecule localization events, with a 1.2-fold increased average photon number, were observed.

As a proof of principle, we fused tdEosFP to the C-terminus of Num1. This fusion does not affect the function of Num1 because replacement of the native *num1* gene with a *num1:tdEosFP* fusion construct does not cause the phenotypic alterations accompanying a non-functional Num1 protein, such as altered hyphal morphology or changed motility of EEs. Thus, tdEosFP appears to be a very feasible tool for super-resolution localization microscopy in *U*. *maydis*.

Num1 has been shown to function in the spliceosome-associated NTC-complex as an SPF27 homologue, and, in accordance with this function, the predominant localization of Num1 is within the nuclear compartment. In our previous studies^14^, we observed an interaction of Num1 with several cytoplasmic proteins including the microtubule-dependent motor protein Kin1, which suggested an additional localization of Num1 within the cytoplasm. Faint cytoplasmic emission was observed from Num1:GFP fusion proteins; however, precise localization of the weak signals was not possible. PALM microscopy with the newly established tdEosFP construct now clearly supports both cytoplasmic localization of Num1 as well as Kin1-dependent movement of Num1. Deletion of the *num1* gene has been shown to affect the transport of EEs. However, we can exclude that the observed motility of Num1 depends on EEs because (1) we did not observe co-localization of Num1:tdEosFP with the endosomal marker Rab5a, and (2) motility of Num1 in hyphae is affected by deletion of *kin1* but not *kin3*. Kin3 is the motor protein required for the plus-end directed transport of EEs on microtubules^4,5^. Thus, if Num1 movement would result from its association with EEs, we would expect an impact of the *kin3* deletion also on Num1 motility. Kin1 is indirectly involved in EE motility via the retrograde transport of dynein; Kin1 activity leads to an accumulation of dynein at a dynein loading zone in the hyphal tip^6^. Since deletion of *kin1* or *num1* has similar phenotypes with respect to dynein mislocalization^6,14^, we have to assume that Num1 affects the motility of EEs more likely by disturbing the kinesin1/dynein transport machinery. In *Saccharomyces cerevisiae*, the transport of dynein via the kinesin motor Kip2 is facilitated by Pac1/LIS1, which acts as a processivity factor for kinesin to suppress hindrance by dynein moving in the opposite direction^43^. Off-loading of dynein is mediated by yeast Num1p by relieving Pac1/LIS1-mediated inhibition. Of note, the yeast Num1p protein shares only its name with the *U*. *maydis* Num1 protein, but shows no homologies^44^. Similar to yeast Num1p, the *U*. *maydis* Num1 protein could also function to establish or maintain the dynamics of a Kin1/Dyn complex. In hyphae, the minus-ends of microtubules accumulate around the nucleus; thus, one would expect the function of a protein that “reverses” the direction of a Kin1/Dyn complex within this region. This would be in accordance with our observation that (a) Num1 is predominantly localized in the vicinity of microtubules within this region (Fig. 3d,b) Num1 motility is mostly observed within this region (Fig. 5a).

As motility and localization of Num1 depend on both the integrity of the cytoskeleton and on Kin1, we have to infer a microtubule-associated localization of Num1. Although we did not detect a clear co-localization with GFP:Tub1, the density of Num1:tdEosFP appears enhanced around microtubules, perhaps due to a weak interaction with microtubule-bound Kin1. Indeed, the interaction between the two proteins appears to be rather weak, as indicated by the observation that co-purification of Num1 and Kin1 is only possible after crosslinking^14^. Such a weak interaction would be in line with a transient function of Num1 in controlling the dynamics of a Kin1/Dyn complex on microtubules.

In ∆*kin1* deletion strains, localization and motility of Num1 are restricted to the nucleus and to a region very close to the nucleus, suggesting that Kin1 is intimately associated with the transfer of the protein from the nucleus to the cytoplasm. Kin1 has been described to interact with the nuclear pore complex^45^, It is well possible that the initial interaction of Num1 and Kin1 appear at this stage, and that Num1 stays attached close to the nuclear envelope without Kin1.

Num1 functions as a component of the splicing-associated NTC^14^. Within *U*. *maydis* hyphae, mRNA is tethered to EEs mediated by the RNA-binding protein Rrm4^3^. The functional connection between nuclear export and loading on EEs of mRNA molecules is currently not yet understood. Similar to what has been described for proteins of the exon junction complex and for the *Drosophila oskar* mRNA reviewed in^46^, one could imagine that Num1 remains attached to mRNA molecules after splicing. An interaction of Kin1 with Num1 at the nuclear pore complex could then be involved in loading of mRNAs to EEs, which would be in agreement with the observed movement of Num1 close to the nucleus. The distinct localization in *∆kin1* strains could be attributed to the tethering of Num1 to the nuclear pore complex, the minimal movement reflecting the dynamic movement of the nuclear envelope (or the nuclear pore complexes).

With the fusion of an optimized tdEosFP to Num1, we have shown the feasibility of this construct for PALM microscopy in *U*. *maydis* by proving the cytoplasmic localization and the Kin1 dependent motility of a spliceosome-associated protein. In general, localization-based super-resolution microscopy using pseudo-monomeric EosFP tandem fusion proteins appears as a promising approach for cell biology in *U*. *maydis* and other fungal systems.

## Materials and Methods

### DNA procedures

Molecular methods followed established protocols^47^. *U*. *maydis* transformation procedures were performed as previously described^48^. PCR-generated linear DNA was used for transformation of *U*. *maydis*^49^. Homologous integration of all constructs was verified by gel blot analysis.

DNA from *U*. *maydis* was prepared as follows: 1.8 ml of an overnight culture was transferred into a 2 ml reaction tube with ≈0.1 ml glass beads (400–850 µm). Cells were precipitated by centrifugation (1 min, 13.000 rpm); the pellet was dissolved in 500 µl lysis buffer (30 mM Tris-HCl pH 7,5, 25 mM Na_2_-EDTA, 0.5% (w/v) SDS). Cells were broken by shaking the samples for 10 min at 1400 rpm on a vibrax shaker (IKA, Staufen, Germany). Samples were incubated at 65 °C for 15 min, chilled on ice for 5 min; 8 M potassium acetate was added before vortexing. After centrifugation (15 min, 13.000 rpm) the supernatant (500 µl) was transferred to a new tube and mixed with 400 µl isopropyl alcohol for DNA precipitation (15 min, 13.000 rpm). DNA was washed once with 70% EtOH (5 min, 13.000 rpm), the pellet briefly air-dried and redissolved in 50 µl 1 × TE containing RNase (50 µg/ml) by incubation at 50 °C for 10 min.

### Plasmid constructions

For construction of tandem dimeric EosFP, we followed the strategy described in^31^. The open reading frame (ORF) for *eos* was di-codon optimized^37^ for *U*. *maydis* based on the EosFP amino acid sequence (GenBank accession: AAV54099.1) using an online tool developed by Florian Finkernagel, IMT, Marburg, Germany (http://dicodon-optimization.appspot.com) and synthesized by Eurofins Genomics (Ebersberg, Germany). The synthesized EosFP ORF harbors a mutation (corresponding to T158R) to prevent tetramerization; nucleotides corresponding to amino acid positions 220 and 108 were altered by silent mutagenesis to delete internal *Bsa*I and *Nco*I sites that would interfere with subsequent cloning. The first copy of EosFP (T158R) was PCR amplified with di-codon optimized EosFP DNA as template using primers P1 and P2 (see Suppl. Table 1) to add terminal 5′ *Sfi*I and 3′ *Bsa*I sites and to delete the STOP codon of the EosFP ORF. The second copy of EosFP (T158R) was generated by PCR amplification using the same template by primer pair P3 and P4 (see Suppl. Table 1); P3 adds a *BsaI* site and a 36 nt linker sequence corresponding to the amino acid sequence GHGTGSTGSGSS 5′ to the tdEosFP ORF; P4 adds a *Asc*I site 3′ of the STOP codon.

The type IIS restriction enzyme *BsaI* cleaves outside of its recognition sequence (in primers P2 and P3), creating four base flanking overhangs that were customized to allow the direct assembly of the two tdEos fragments. The fusion via *BsaI* resulted in a tandem arrangement of two EosFP monomers linked by the sequence GHGTGSTGSGSS (tdEosFP). The sequence of the tdEosFP protein is given in Suppl. Fig. 1. The SfiI/AscI fragment harbouring the tdEosFP ORF was subsequently exchanged with the *SfiI/AscI* fragment harboring the eGFP gene in plasmid pUMa317^50^. In the resulting plasmid pBL7, the tdEosFP ORF is flanked at the 3′ end by the *nos* terminator, followed by a hygromycin-resistance cassette (*hyg*^*R*^) to allow selection in *U*. *maydis* (Suppl. Fig. 2). The entire tdEosFP/hyg^R^ fragment is bordered by two *SfiI* sites that allow fusion of DNA-fragments for homologous recombination in *U*. *maydis* in a single step^49,50^.

The *U*. *maydis* di-codon optimized *mEos2* gene was synthesized by Geneart (Regensburg, Germany) and integrated via *Sfi*I (5′ end)/*Asc*I (3′ end) into pUMa317 (pMF5-1h,^51^).

Plasmid pPtefRab5aGn-Cbx^R^ harbors an N-terminal fusion of the *eGFP* open reading frame to *rab5a* Rab5a (UMAG_10615; GenBank accession XP_011387349.1) under control of the tef-promoter^52^, and a carboxin cassette for the integration into the *ip*-locus^53^ (M. Feldbrügge, University of Düsseldorf, Germany, unpublished).

### Strains and growth conditions

*Escherichia coli* strain TOP10 (Invitrogen, Carlsbad, USA) was used for cloning purposes. Growth conditions and media for cultivation were followed as described previously^47^. *U*. *maydis* cells were grown in CM Complete Medium^54^, containing 1% glucose (CM-G) or 1% arabinose (CM-A), respectively, at 28 °C. Solid media contained 2% agar. For microscopic analyses, cells were taken from logarithmically growing liquid cultures in CM-G medium. For the investigation of hyphae, cells were transferred from CM-G to CM-A and induced for 6 h, as described^38^. 200 µl cells were mixed with 200 µl 4% low melting agarose in H_2_0 (45 °C) in 8 well chambers (μ-Slide 8 well, ibidi GmbH, Munich, Germany) and analyzed immediately.

#### U. maydis strains used in this study were generated as follows

AB31 *num1:tdEosFP*: To generate a tdEosFP fusion to Num1 (UMAG_01682, GenBank XP_011387660.1) 1 kb fragments corresponding (a) to the *num1* ORF (primers P5, P6, Suppl. Table 1) and (b) to the genomic 3′ region of *num1* (primers P7, P8, Suppl. Table 1) were generated by PCR, digested with *SfiI* (added by primers P6 and P7, respectively) and ligated to the SfiI-digested *tdEosFP:hyg*^*R*^ cassette from plasmid pBL7. The ligation product was PCR amplified and integrated to the native *num1* locus of strain AB31^38^ via homologous recombination, following previously described protocols^49^.

AB31 *num1:mEos2*: To generate a *mEos2* fusion to Num1, integration of the *mEos2*:*hyg*^*R*^ cassette into AB31 was performed as described for *num1:tdEosFP:hyg*^*R*^.

AB31 *num1:tdEosFP GFP:Tub1:* Plasmid P_*otef*_*:gfp:tub1* (*tub1*: UMAG_01221; GenBank XP_011387157.1) for ectopic expression of a GFP:tubulin fusion protein under control of the constitutive *otef*-promoter^40^ was targeted in single copy into the *ip-l*ocus (*cbx*-locus)^54^ of AB31 *num1:tdEosFP*.

AB31 *num1:tdEosFP* Rab5a:GFP: Plasmid pPtefRab5aGn-Cbx^R^ for ectopic expression of a Rab5a:eGFP fusion protein was targeted in single copy into the *ip-l*ocus (*cbx*-locus)^53^ of AB31 *num1:tdEosFP*.

AB31 *num1: tdEosFP∆kin1;* AB31 *num1:tdEosFP∆kin3::* The open reading frame of *kin1* or *kin3* was replaced in AB31 *num1:tdEosFP* by the nourseothricin resistance cassette from pUMa262^50^ using the PCR-based protocol for gene replacements previously described^49^. Flanking regions for deletion of *kin1* (UMAG_04218; GenBank XP_011390699.1) were generated by PCR using primer pairs P15/P16 and P17/P18 (Suppl. Table 1) on genomic *U*. *maydis* DNA as template. For deletion of *kin3* (UMAG_06251; GenBank XP_011392595.1) primer pairs P19/P20 and P21/P22 (Suppl. Table 1) were used. Homologous integration was verified by Southern Blot Analysis for all strains generated within this study. Genotypes for all *U*. *maydis* strains used in this study are listed in Table 1.

**Table 1:** Strains used in this study.

Strain	Genotype	Reference
AB31	*a2*, P_crg1_: *b*W2, P_crg1_:*b*E1 *ble*^*R*^	^38^
AB31 *num1:tdEosFP*	*a2*, P_crg1_:*b*W2, P_crg1_:*b*E1 *ble*^*R*^, *num1:tdEosFP hyg*^*R*^	This study
AB31 *num1:mEos2*	*a2*, P_crg1_:*b*W2, P_crg1_:*b*E1 *ble*^*R*^, *num1:mEos2:hyg*^*R*^	This study
AB31 *num1:tdEosFP* Rab5a:GFP	*a2*, P_crg1_:*b*W2, P_crg1_:*b*E1 *ble*^*R*^, *num1:tdEosFP hyg*^*R*^, *ip*^*r*^*[P*_*otef*_*:gfp:rab5a]ip*^*s*^	This study
AB31 *num1: tdEosFP∆kin1*	*a2*, P_crg1_:*b*W2, P_crg1_:*b*E1 *ble*^*R*^, *num1:tdEosFP hyg*^*R*^,*∆kin1::nat*^*R*^	This study
AB31 *num1: tdEosFP∆kin3*	*a2*, P_crg1_:*b*W2, P_crg1_:*b*E1 *ble*^*R*^, *num1:tdEosFP hyg*^*R*^, *∆kin3::nat*^*R*^	This study
AB31 *num1:tdEosFP GFP:Tub1*	*a2*, P_crg1_:*b*W2, P_crg1_:*b*E1 *ble*^*R*,^, *num1:tdEosFP:hyg*^*R*^, *ip*^*r*^*[P*_*otef*_*:gfp:tub1]ip*^*s*^	This study

Abbreviations: *a*, *b*: mating type loci; *∆*: deletion; P: promoter;::, homologous replacement;:, fusion; *hyg*^*R*^: hygromycin resistance; *ble*^*R*^: phleomycin resistance; *nat*^*R*^, nourseothricin resistance; *gfp*: green fluorescent protein; *tub1*: α-tubulin, *ip*^*r*^*[xxx]ip*^*s*^: integration of construct in the *ip*-locus resulting in carboxin resistance.

### Benomyl treatment and fixation

For disruption of the microtubule cytoskeleton, 2 µl benomyl (10 mM in DMSO) were added to 500 µl cells to obtain a final concentration of 40 µM and mixed gently. After 5-10 min incubation at room temperature, microtubules were completely disintegrated. For fixation of cells, 500 µl culture was mixed with 500 µl fixative solution (50 ml 200 mM PIPES pH 6.7, 10 ml 500 mM EGTA pH 8.5, 1 ml 1 M MgSO_4_, 10 ml DMSO, 21,6 ml 37% formaldehyde) and incubated for 30 min on a spinning wheel at room temperature. Cells were washed once in 1× PBS and dissolved in 500 µl (final concentration 8% formaldehyde).

### Cell imaging

Fluorescence and DIC images: (1) Axioimager Z1 microscope equipped with an Axiocam MRm camera (Carl Zeiss, Jena, Germany). Standard filter sets (Green: 38 HE, excitation 470/40, emission 525/50; Red: 43 HE, excitation 550/25; emission 605/70; DAPI: 49, excitation 365, emission 445/50; all filter sets from Carl Zeiss, Jena, Germany) were used for epifluorescence analysis. EosFPs were photoconverted with the DAPI filter set with a wavelength maximum of 365 nm in 20 s intervals. After each interval, red and green emitting species were imaged with red and green filter sets, respectively, with 600 ms exposure time (2) Andor Revolution XD spinning disk confocal laser scanning microscope (BFi OPTiLAS, München, Germany) with an OLYMPUS ApoN60×/1.49 oil immersion objective. Cells were imaged with a 488 nm laser to excite mEos2 or tdEosFP in the green channel and filtered with a 525/50 (peak wavelength/width) band pass filter (Semrock, New York, NY); images were further analyzed with ImageJ.

Localization microscopy images were acquired with a custom-built widefield microscope with two-channel detection. The system is based on an Axio Observer Z1 (Zeiss, Göttingen, Germany) frame, equipped with a C-Apochromat 63/1.2 W Corr objective, an EMCCD camera (Ixon Ultra 897, Andor, Belfast, UK) and multiple excitation lasers including a 561 nm laser (Gem 561, Laser Quantum, Konstanz, Germany), a 473 nm laser (LuxX 473-100, Omicron-Laserage Laserprodukte, Rodgau-Dudenhofen, Germany) and a 405 nm laser (Stradus 405-250, Vortran Laser Technology, Sacramento, CA). Details of the apparatus have been published elsewhere^55^.

The fusion proteins Num1:mEos2 and Num1:tdEosFP were photoconverted to their red emitting species by 405 nm laser irradiation with 0–50 W cm^−1^ laser intensity and simultaneously excited by a 561 nm laser (200–400 W cm^−1^). A 607/70 band-pass filter (Semrock, New York, NY) was used to filter the fluorescence emission, which was detected by an EMCCD camera with 50 ms exposure time for 1000–1500 frames. Molecule localization and image calculations were performed with the custom-written analysis software a-livePALM^30^ running under the MATLAB R2015b (The Mathworks, Natick, MA) environment. Within the software, the photon threshold was set to 100 for Figs 1, 2A (left) and Fig. 4B, and 300 for all other PALM images.

For images in two color channels, we first excited the cells with a 473 nm laser (100–200 W cm^−1^). Typically, 100 continuous frames were taken to image the emission from GFP:Tub1 and GFP:Rab5, filtered with a 512/25 band pass filter (Semrock, New York, NY). After a delay of typically 3 s to change filters, we imaged Num1:tdEosFP as described above. We used a-livePALM to generate localization microscopy images for the data in the red channel and merged them with images in the green channel created by using ImageJ.

Kymographs were generated with the ImageJ software^56^ using a Kymograph Plugin (https://www.embl.de/eamnet/html/body_kymograph.html) along a straight line tracing the long axis of the whole cell. The line width was set equal to the width of the cell to cover the entire cell, so the intensity was integrated perpendicular to the long axis. All kymographs were generated from image stacks with 500 frames.

## Electronic supplementary material

Supplementary Material
