# Supplementary material for: Cytoplasmic Transport Machinery of the SPF27 Homologue Num1 in Ustilago maydis
Source: Sci Rep. 2018 Feb 26;8:3611. doi: 10.1038/s41598-018-21628-y (PMC5832149; doi:10.1038/s41598-018-21628-y)
Supplement: Supplementary file 1 — Supplementary Material [file 41598_2018_21628_MOESM1_ESM.pdf]

Supplementary Online Materials

Cytoplasmic Transport Machinery of the SPF27 Homologue Num1 in *Ustilago maydis*

Lu Zhou, Theresa Obhof, Karina Schneider, Michael Feldbrügge, G. Ulrich Nienhaus, Jörg Kämper

Supplementary Table 1: Oligonucleotides used in this study

	Oligonucleotide	Sequence (5' – 3')
**Primers used for construction of tdEosFP**		
P1	eosfor-sfiI	GTTGGCCAACGCGGCCATGGGCGCCATCAAGCC
P2	eosrev1	GCTTCACGGTCTCGACCgcgacgagcgttgtcggg
P3	eosfor2	GCTTCACGGTCTCCcGGTCACGGCACCGGCTCGACCGGCTCGGGCTCGTCG
P4	eosrev-ascI	GGCGCGCCGTTGCGGCCGCTTAGCGACGA
**Primers used for Num1 fusion (*num1, umag_01682)***		
P5	01682GFP_LB_se	CCAATTCTTAACGGCCGAAAAGAC
P6	01682GFP_LB_as	GTGGGCCGCGTTGGCCCGCTCCAACAGGCTCAACTC
P7	01682GFP_RB_Sfi	CACGGCCTGAGTGGCCTAGGCAGTGATGCGGTTCAT
P8	01682GFP_RB_as	GACGGCATCGTTGCGTTGGAAG
**Primers used for gene deletions**		
	kin1 (umag_04218)	
P15	Kin1 LB_se	GTGTGCTTTGTGGAGGAAGTCCGAC
P16	Kin1 LB_as	gttggccatctaggccGACGACTTGGCGGTGTAAGTGTGC
P17	Kin1 RB_se	gttggcctgagtggccCGTGGAAGATCTATACGTTTCACCTT
P18	Kin1fusion_RB_as	GCAATCTCAAGCGTCGCTATGA
	kin3 (umag_06251)	
P19	Kin3_LB_se_new	GAACTGCTCCAAAACTCGTGACTCG
P20	Kin3_LB_as_new	CATggccatctaggccGATGGCGGAGCTCTATGGAGGT
P21	Kin3_RB_se	CATggcctgagtggcc ATTCTAGAAGGCGTCATGGCCAAA
P22	Kin3_RB_as	ACGAATACGCTCGTGGCTAGGT

>tdEosFP
MGAIKPDMKINLRMEGNVNGHHFVIDGDGTGKPFEGKQSMDLEVKEGGPLPFAFDILT
TAFHYGNRVFAEYPDHIQDYFKQSFPKGYSWERSLTFEDGGICIARNDITMEGDTFYNK
VRFHGVNFPANGPVMQKKTLKWEPSTEKMYVRDGVLTGDIRMALLLEGNAHYRCDFR
TTYKAKEKGVKLPGYHFVDHCIEILSHDKDYNKVKLYEHAVAHSGLPDNARRghgtgstgs
gssMGAIKPDMKINLRMEGNVNGHHFVIDGDGTGKPFEGKQSMDLEVKEGGPLPFAFDI
LTTAFHYGNRVFAEYPDHIQDYFKQSFPKGYSWERSLTFEDGGICIARNDITMEGDTFY
NKVRFHGVNFPANGPVMQKKTLKWEPSTEKMYVRDGVLTGDIRMALLLEGNAHYRCD
FRTTYKAKEKGVKLPGYHFVDHCIEILSHDKDYNKVKLYEHAVAHSGLPDNARR

Supplementary Figure 1: Sequence of tdEosFP. The linker sequence tethering the two EosFP open reading frame is given in lower-case letters, the amino acids (T158R) that were altered to prevent tetramerization are underlined.

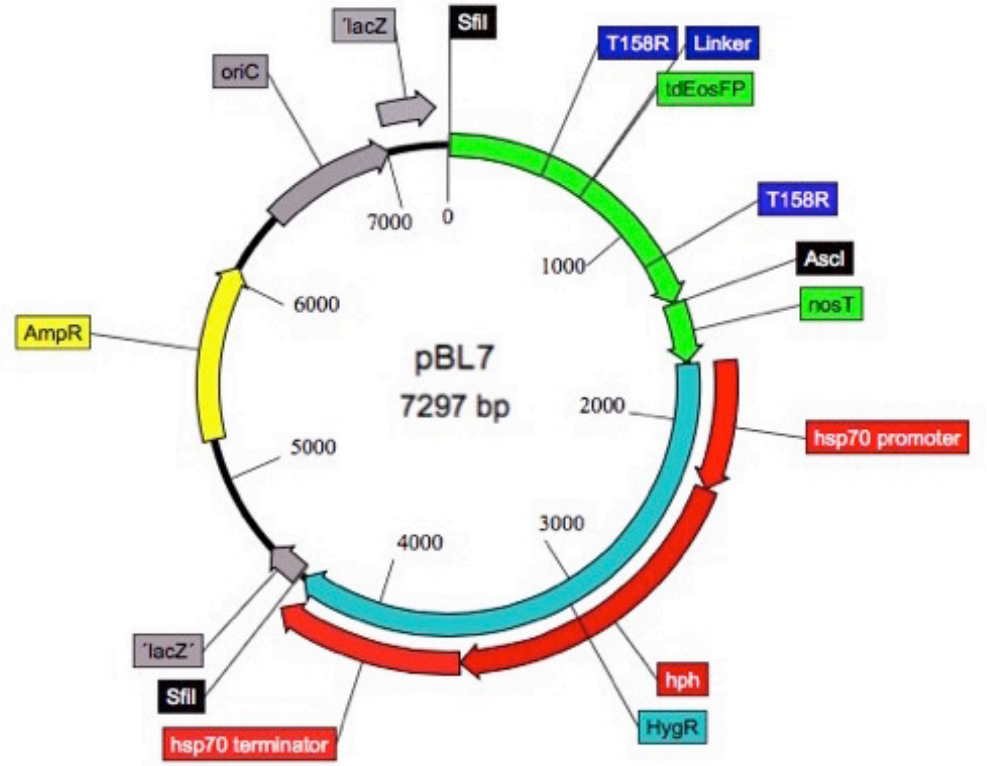

Supplementary Figure 2: Map of plasmid pBL7. The *Sfi*I fragment harbors the gene for the tandem dimeric Eos fluorescent protein (tdEosFP) and a hygromycin resistance cassette for selection in *Ustilago maydis*. DNA-fragments for homologous recombination can be fused to the tdEosFP/hyg^{R} cassette via the 5´and 3´ SfiI sites. Sequence of the linker between the two Eos open reading frames of tdEosFP: GHGTGSTGSGSS.
